# Body size over the adult life course and the risk of colorectal cancer among postmenopausal women

**DOI:** 10.1017/S1368980023000988

**Published:** 2023-08

**Authors:** Le Su, Michael Hendryx, Ming Li, Aladdin H Shadyab, Nazmus Saquib, Marcia L Stefanick, Juhua Luo

**Affiliations:** 1 Department of Epidemiology and Biostatistics, School of Public Health, Indiana University – Bloomington, Bloomington, IN 47408, USA; 2 Department of Environmental and Occupational Health, School of Public Health, Indiana University, Bloomington, IN, USA; 3 Department of Family Medicine and Public Health, School of Medicine, University of California San Diego, San Diego, California, USA; 4 College of Medicine at Sulaiman Al-Rajhi University, Al Bukayriyah, Kingdom of Saudi Arabia; 5 Stanford Prevention Research Center, Stanford University, Stanford, CA, USA

**Keywords:** BMI, Colorectal cancer, Body size, Obesity

## Abstract

**Objective:**

To assess the associations among several anthropometric measures, as well as BMI trajectories and colorectal cancer (CRC) risk in older women.

**Design:**

Prospective cohort study.

**Setting:**

Forty clinical centres in the USA.

**Participants:**

Totally, 79 034 postmenopausal women in the Women’s Health Initiative Observational Study.

**Results:**

During an average of 15·8 years of follow-up, 1514 CRC cases were ascertained. Five BMI trajectories over 18–50 years of age were identified using growth mixture model. Compared with women who had a normal BMI at age 18, women with obesity at age 18 had a higher risk of CRC (HR 1·58, 95 % CI 1·02, 2·44). Compared with women who kept relatively low normal body size during adulthood, women who progressed from normal to obesity (HR 1·29, 95 % CI 1·09, 1·53) and women who progressed from overweight to obesity (HR 1·37, 95 % CI 1·13, 1·68) had higher CRC risks. A weight gain > 15 kg from age 18 to 50 (HR 1·20, 95 % CI 1·04, 1·40) and baseline waist circumference > 88 cm (HR 1·33, 95 % CI 1·19, 1·49) were associated with higher CRC risks, compared with stable weight and waist circumference ≤ 88 cm, respectively.

**Conclusion:**

Women who have a normal weight in early adult life and gain substantial weight later, as well as those who are persistently heavy over adulthood, demonstrated a higher risk of developing CRC. Our study highlights the importance of maintaining a healthy body weight over the life course for reducing the risk of developing CRC in women.

Colorectal cancer (CRC) is the third most common cancer worldwide and second most common cancer in women^(1)^. Known risk factors of CRC include obesity, diabetes, family history of CRC, inflammatory bowel disease, smoking and excessive consumption of alcohol, red meat and processed meat^(2,3)^. Also, physical activity, precancerous lesions removal, nonsteroidal anti-inflammatory drugs use and hormone therapy are associated with a lower risk of CRC^(2)^.

In recent decades, the relationship between adult obesity as mainly assessed by BMI and CRC risk has been extensively investigated. Higher adult BMI is linked to a higher risk of CRC, with a relatively stronger association in men, whereas no or a weaker association has been found in women^(4)^. In contrast to adult BMI, abdominal adiposity, as determined by waist circumference or waist:hip ratio, is consistently associated with CRC risk in men and women^(5)^.

In addition, given the long latency of colorectal carcinogenesis^(2)^, young adulthood obesity and adult weight changes have been hypothesised to be associated with later risk of CRC. Studies found that obesity in early adulthood (e.g. age 18 years) might be a stronger risk factor than obesity in middle age (e.g. age 50 years) for the development of CRC in women^(6,7)^. Also, studies of the association between weight change (e.g. aged 20–50 years) and CRC risk have yielded inconsistent results^(8–10)^. However, studies focusing on the association between young adulthood obesity, adult weight change and CRC risk are sparse, with limited studies having prospective data with sufficient sample sizes^(6,7,10–14)^.

Weight change, generally derived from two discrete time points, is limited to capturing the body size dynamics across the lifespan. Growth Mixture Model (GMM), however, is a promising approach to studying the cumulative impact of adiposity on several obesity-related diseases, based on classifying individuals into relatively homogeneous body size trajectories^(15)^. Previously, one study investigated the association between body size trajectories based on BMI at age 20, 50 and baseline and CRC risk in the Prostate, Lung, Colorectal, and Ovarian Cancer Screening Trial^(16)^. The current study failed to examine BMI trajectory in women and men separately, while evidence showed that sex difference exists in the association between obesity and CRC risk^(6)^. Understanding whether body size over the adult life course influences is associated with CRC risk among women may help to develop targeted strategies for preventing the disease.

The Women’s Health Initiative Observational Study (WHI-OS) collected data on the body size of women throughout their adult life (retrospectively at ages 18, 35, 50 and at WHI baseline). We utilised WHI-OS to evaluate the associations between anthropometric measures, including age-specific BMI, BMI trajectory, weight gain and waist circumference with the risk of CRC.

## Material and methods

### Study population

The Women’s Health Initiative is a prospective cohort study that aimed to investigate the risk factors associated with the morbidity and mortality of postmenopausal women. Women aged 50–79 years were recruited throughout forty clinical centres in the USA from 1993 to 1998. Greater detail on the study design and data collection has been published elsewhere^(17)^. All participants provided written informed consent, and the study was approved by the institutional review boards at each clinical centre.

**Fig. 1:**
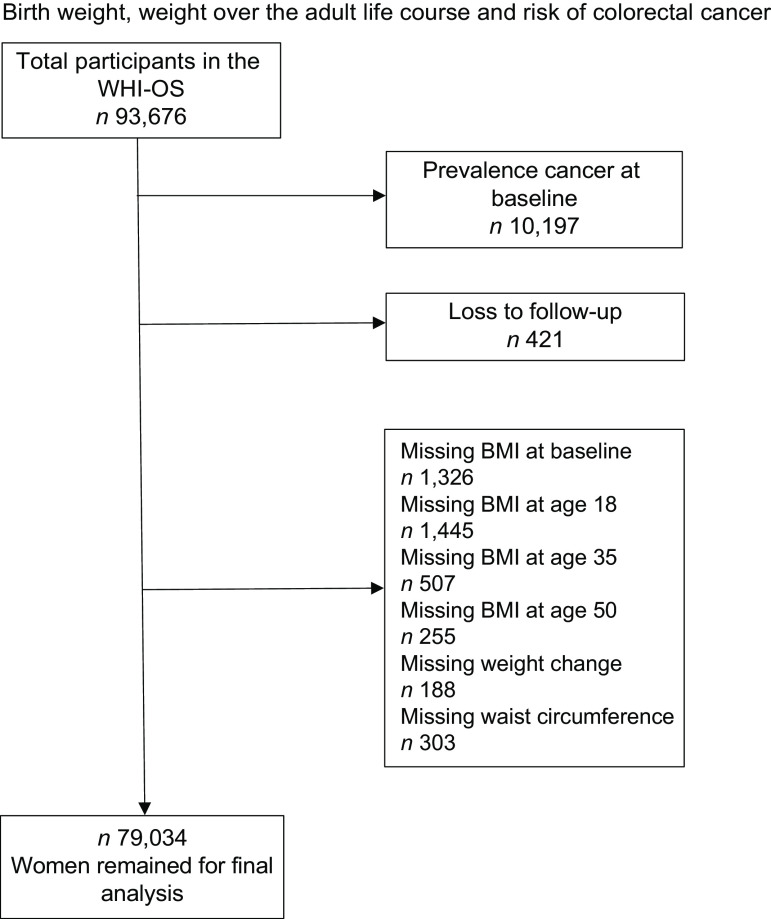
Flow diagram of participants included in the analysis

Among a total of 93 676 women in WHI-OS, we excluded those who reported cancer history (except nonmelanoma skin cancer) at baseline (*n* 10 197); women without follow-up information (*n* 421) and women without data for main exposure (*n* 4024), including missing BMI at baseline (*n* 1326), missing BMI at age 18 (*n* 1445), missing BMI at age 35 (*n* 507), missing BMI at age 50 (*n* 255), missing weight change between 18 and 50 (*n* 188) and missing waist circumference (*n* 303). The final analytic sample included 79 034 women (Fig. 1).

### Exposure

#### BMI at age 18, 35 and 50 and at Women’s Health Initiative baseline

Self-reported heights and weights at ages 18, 35 and 50 were collected at study entry in the WHI-OS^(17)^. In addition, weight and height at baseline were measured by certified staff at clinic visits with a balance beam scale and a wall-mounted stadiometer. The corresponding BMI were computed as weight (kg) divided by the square of the measured height (cm^2^). According to WHO categories, underweight, normal weight, overweight and obesity were defined as a BMI of less than 18·5 kg/m^2^, 18·5–24·9 kg/m^2^, 25–29·9 kg/m^2^ and 30 kg/m^2^ or greater, respectively.

#### BMI trajectory

**Fig. 2:**
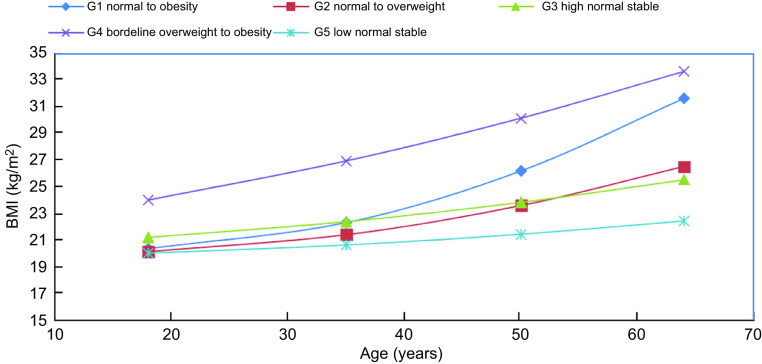
Five BMI trajectories using Growth Mixture Model

We identified BMI (in continuous) trajectories that represent varying adiposity change patterns through ages 18, 35, 50 and WHI baseline by using GMM (Mplus software version 8·8)^(18)^. We allowed the model to infer 2–5 potential trajectories as latent classes based on linear or quadratic BMI patterns. The optimal model was selected based on the model fit indices (Bayes Information Criteria and Akaike information criteria) and the class size (more than 5 % of participants for each group) (online Supplementary Table 1). In addition, the interpretability of each class trajectory is considered by plotting each class trajectory. For this purpose, each class trajectory should be distinct and separate from each other. Five BMI trajectory categories with quadratic patterns were selected, representing low normal stable, high normal stable, normal to overweight, normal to obesity and borderline overweight to obesity (Fig. 2). Women were assigned to different trajectory groups based on their highest posterior probabilities. Noted, these potential BMI trajectories did not represent accurately body size profiles for each person, but rather approximations of true development patterns.

#### Weight changes

Weight change between 18 and 50 years was derived by subtracting weight at age 18 from weight at age 50. Weight change between 18 and 50 was approximate normal distribution (mean: 9·0 kg, sd: 9·19 kg). Thus, we chose four categories based on weight change distribution, as well as consideration of conventional cut-points. They are weight loss (< –5 kg), stable weight (–5 kg to 5 kg), weight gain (5–15 kg) and excessive weight gain (> 15 kg).

#### Waist circumference

Waist circumference was measured at baseline using tape measures at the narrowest part of the torso. A high waist circumference was defined as > 88 cm^(19)^.

### Ascertainment of cases

The cancer ascertainment and adjudication methods in WHI have been previously reported^(20)^. Briefly, colorectal cancer incidents were initially collected by a mailed self-administered Medical History Update and then confirmed by trained physician adjudicators after reviewing medical records, histologic and pathology reports during follow-up. After centralised review, the cancer was coded by trained cancer coders based on the Surveillance Epidemiology and End Results guidelines^(20)^.

#### Covariates

Demographic and behavioural characteristics, as well as family and medical history information, were collected at baseline using self-reported standardised questionnaires. Based on literature review and available information collected at baseline, the following variables were considered as potential confounders^(2)^: age (in continuous), race/ethnicity (Asian or Pacific Islander, American Indian or Alaska Native, Black, Hispanic/Latina, non-Hispanic white and other), education (high school or less, some college/technical training, college degree or higher), physical activity (total metabolic equivalent tasks (MET)-hours per week), pack-years of smoking (never, < 5, 5–20, ≥ 20), alcohol use (nondrinker, past drinker, current drinker with < 7 drinks/week, current drinker with ≥ 7 drinks/week), healthy eating index score (in continuous), family history of CRC (yes/no), CRC screening (never, < 5 years ago, ≥ 5 years ago), history of colorectal polyp removal (yes/no), diabetes (yes/no), prior hormone use (none, estrogen alone, oestrogen and progestin, mixed) and nonsteroidal anti-inflammatory drugs use (yes/no). In addition, healthy eating index (HEI) was also included as a covariate. HEI, which was created by the U.S. Department of Agriculture and the National Cancer Institute, reflects an integrated picture of diet quality^(21)^. HEI scores were computed from the FFQ, which was developed by WHI dietary assessment working group^(22)^. A higher HEI score represents a better alignment with dietary recommendations.

### Statistical analysis

Descriptive statistics were used to summarise demographic and behavioural characteristics at baseline between participants with or without CRC. *χ* ^2^ tests and two-sample *t* tests were used to examine the differences between participants with CRC and participants without CRC for categorical and continuous variables, respectively. For non-normally distributed variables, the Mann–Whitney tests were used. GMM model was used for modelling trajectories of BMI.

Cox proportional hazards regression was utilised to estimate hazard ratios (HR) with corresponding 95 % CI as the effect of age-specific BMI, BMI trajectories, waist circumference and adult weight gain from age 18 to 50, on the risk of CRC. BMI at different ages, baseline waist circumference and adult weight change were evaluated as both categorical and continuous variables. In the multivariable-adjusted models, covariates as mentioned above have been adjusted, including age, race/ethnicity, education, physical activity, pack-years of smoking, alcohol use, healthy eating index score, family history of CRC, CRC screening, history of colorectal polyp removal, diabetes, prior hormone use and NSAID use. Height was adjusted for adulthood weight change in the model. The proportional hazard assumptions were tested by adding interaction terms between the covariates and the survival time to the model and assessing their significance using the likelihood ratio test. Proportional hazard assumptions hold for the covariates in the model, since all interaction terms are not statistically significant (the smallest *P* value is 0·06).

Effect modifications by physical activity, family history of CRC, education, alcohol consumption, smoking status and hormone therapy were evaluated for all exposures using the likelihood ratio tests. In sensitivity analysis, to evaluate potential reverse causation due to pre-existing disease, we repeated our analysis by excluding the first two years of follow-up. We took possible misclassification of trajectory membership into account by using the posterior probabilities as the exposure (except the reference) in the multivariable-adjusted model. We also assessed whether the association between BMI trajectories/ waist circumference at baseline and risk of CRC varied by cancer subtypes (colon cancer or rectum cancer). The associations between age-specific BMI/weight changes and rectum cancer risks were not present, since the number of cases (≤ 5) for rectum cancer was too small after stratified by BMI categories or weight change categories.

Statistical analyses were conducted using SAS version 9.4 (SAS In statute). *P*-values were two-sided.

## Results

There was a total of 1514 incident CRC cases among 79 034 women during an average of 15·8 years of follow-up. Table 1 presents the demographic and lifestyle characteristics of cases and non-cases at baseline. Compared with women without CRC, women who developed CRC during follow-up were more likely to be older, non-Hispanic White, heavier smokers, less frequently have CRC screening, more likely to have a family history of CRC, more likely to have polyp removal or more likely to be never hormone users. Also, they were more likely to have higher BMI at different ages and higher waist circumference at baseline.

**Table 1:** Baseline characteristics of colorectal cancer cases *v*. non-cases

Variables	Non-case participants			Colorectal cases			*P* value
	%	Mean	sd	%	Mean	sd	
Total number of women	77 520			1514			
Age at baseline*		63·49	7·34		65·67	6·94	< 0·01
Race/Ethnicity (%)†							
White	84·10			87·23			< 0·01
Black	7·58			7·35			
Hispanic	3·75			1·92			
American Indian	0·39			0·20			
Asian/Pacific Islander	3·05			2·12			
Others	1·13			1·19			
Education (%)†							
High school diploma	21·07			19·55			0·07
School after high school	36·32			39·10			
College degree or higher	42·61			41·36			
Physical activity (MET-hours/week)‡							
Median		10·00			9·50		0·06
IQR		3·50, 20·08			3·00, 18·92		
Total energy intake (kcal/d)*	1522·2		599·5	1526·9		600·3	0·77
HEI 2015*	67·21		10·25	66·83		10·49	0·15
Pack years of smoking (%)†							
Never smoker	52·47			49·52			< 0·01
< 5	14·85			12·64			
5–20	14·41			13·94			
≥ 20	18·27			23·90			
Alcohol use (%)†							
Non-drinker	11·12			9·82			0·33
Past drinker	18·28			17·65			
< 7 drinks per week	57·79			59·12			
7+ drinks per week	12·80			13·40			
Family history of colorectal cancer (%)†							
Yes	15·07			19·09			< 0·01
No	84·93			80·91			
Date of last colonoscopy†							
None	46·35			50·00			< 0·01
< 5 years ago	34·50			30·24			
≥ 5 years ago	19·15			19·76			
History of colorectal polyp removal (%)†							
Yes	17·37			22·05			< 0·01
No	82·63			77·95			
Diabetes (%)†							
Yes	5·09			6·21			0·05
No	94·91			93·79			
Non-steroidal anti-inflammatory drugs use (%)†							
Yes	5·67			5·55			0·84
No	94·33			94·45			
Prior hormone use (%)†							
None	38·69			48·15			< 0·01
Estrogen alone	31·25			27·87			
Estrogen plus progestin	23·75			18·49			
Mixed	6·31			5·48			
Adulthood body size							
BMI at age 18 (kg/m^2^)*		20·82	2·68		21·01	2·67	< 0·01
BMI at age 35 (kg/m^2^)*		22·24	2·93		22·46	2·90	< 0·01
BMI at age 50 (kg/m^2^)*		24·19	3·96		24·46	3·89	< 0·01
BMI at baseline (kg/m^2^)*		26·97	12·80		27·63	5·58	< 0·01
Height at age 18 (cm)*		163·62	6·58		164·16	6·51	< 0·01
Height at baseline (cm)*		161·75	6·39		162·10	6·35	0·04
Weight change between 18 and 50 (kg)*		9·02	9·21		9·29	9·39	0·25
Waist circumference at baseline (cm)*		84·08	12·80		86·71	13·10	< 0·01

IQR, interquartile range; HEI, healthy eating index; MET, metabolic equivalent of task; kcal, kilocalories; kg, kilogram; cm, centimetre; m, metre.

Results are presented by means ± sd, medians (interquartile range) or proportion.

* Two-sample *t* test.

† *χ* ^2^ test.

‡ Mann–Whitney test.

### Age-specific BMI and risk of colorectal cancer

Compared with women with normal BMI at age 18, women with obesity at age 18 had a higher risk of CRC (HR 1·58, 95 % CI 1·02, 2·44). The risk of CRC was also elevated among women with overweight (HR 1·35, 95 % CI 1·20, 1·52) or with obesity (HR 1·30, 95 % CI 1·07, 1·59) at age 50 relative to women with normal BMI at age 50. Obesity at WHI baseline was associated with higher CRC risk in women relative to normal weight women at baseline (HR 1·30, 95 % CI 1·14, 1·49). For 5 kg/m^2^ increased in BMI at age 18, 35, 50 and at baseline, the risk of CRC increased ranging from 10 % to 18 % in women (Table 2).

**Table 2:** Body size over the adult life course and risks of colorectal cancer*

	Colorectal cancer cases/No. of participants	Age-adjusted		Multivariable-adjusted†	
		HR	95 % CI	HR	95 % CI
BMI at age 18					
Underweight	219/12 591	0·91	0·79, 1·06	0·91	0·78, 1·05
Normal (reference)	1194/61 270	1(reference)		1(reference)	
Overweight	77/4404	1·00	0·80, 1·27	0·91	0·72, 1·15
Obesity	21/769	1·71	1·11, 2·63	1·58	1·02, 2·44
Hazard ratio for 5 kg/m^2^ increase	1511/79 034	1·22	1·10, 1·34	1·16	1·05, 1·28
BMI age 35					
Underweight	54/3669	0·77	0·59, 1·02	0·75	0·57, 0·99
Normal (reference)	1249/64 901	1(reference)		1(reference)	
Overweight	167/8467	1·17	1·00, 1·38	1·06	0·89, 1·25
Obesity	41/1997	1·42	1·05, 1·94	1·23	0·90, 1·68
Hazard ratio for 5 kg/m^2^ increase	1511/79 034	1·25	1·15, 1·36	1·18	1·08, 1·29
BMI age 50					
Underweight	28/1601	1·005	0·69, 1·46	1·004	0·69, 1·46
Normal (reference)	926/51 608	1(reference)		1(reference)	
Overweight	425/18 897	1·45	1·29, 1·63	1·35	1·20, 1·52
Obesity	132/6928	1·52	1·27, 1·83	1·30	1·07, 1·59
Hazard ratio for 5 kg/m^2^ increase	1511/79 034	1·26	1·18, 1·34	1·18	1·10, 1·26
BMI at baseline					
Underweight	14/957	0·92	0·54, 1·56	0·91	0·54, 1·56
Normal (reference)	550/32 272	1(reference)		1(reference)	
Overweight	531/27 299	1·16	1·03, 1·31	1·08	0·97, 1·24
Obesity	416/18 506	1·52	1·34, 1·72	1·30	1·14, 1·49
Hazard ratio for 5 kg/m^2^ increase	1511/79 034	1·16	1·10, 1·22	1·10	1·05, 1·16
Weight change between 18–50‡					
Weight loss < –5 kg	43/2087	1·20	0·89, 1·64	1·16	0·85, 1·58
Normal (–5 kg to 5 kg)	518/28 164	1(reference)		1(reference)	
Weight gain (5–15 kg)	636/32 579	1·12	0·99, 1·26	1·08	0·96, 1·22
Weight gain (> 15 kg)	314/16 204	1·38	1·20, 1·60	1·20	1·04, 1·40
Hazard ratio for 5 kg increase	1511/79 034	1·08	1·05, 1·11	1·05	1·02, 1·08
Waist circumference at baseline					
Waist circumference ≤ 88 cm	907/53 156	1(reference)		1(reference)	
Waist circumference > 88 cm	604/25 878	1·49	1·35, 1·66	1·33	1·19, 1·49
Hazard ratio for 5 cm increase	1511/79 034	1·10	1·08, 1·12	1·08	1·06, 1·10

HR, hazard ratio; kg, kilogram; cm, centimeter; CRC, colorectal cancer; NSAID, nonsteroidal anti-inflammatory drugs.

* Cox proportional hazard regression model was used for constructed models.

† In the multivariate-adjusted models, adjusted covariates are age, race/ethnicity, education, physical activity, pack-years of smoking, alcohol use, healthy eating index score, family history of CRC, CRC screening, history of colorectal polyp removal, diabetes, prior hormone use and NSAID use.

‡ Height was further adjusted in weight change model.

### Weight change and risk of colorectal cancer

Weight change during adulthood was associated with CRC risk among postmenopausal women (*P*_trend_ < 0·05). Compared with women with stable weight (±5 kg) from ages 18 to 50, those who gained more than 15 kg had a higher risk of developing CRC. The HR and corresponding 95 % CI were 1·20 (1·04, 1·40). Weight loss and weight gain of less than 15 kg were not significantly associated with CRC. For 5 kg increase in weight change between age 18 and 50, the risk of developing CRC increased 5 % in women (HR 1·05, 95 % CI 1·02, 1·08) (Table 2).

### Waist circumference and risk of colorectal cancer

Waist circumference > 88 cm at baseline was associated with a higher risk of CRC among postmenopausal women (HR 1·33, 95 % CI 1·19, 1·49), compared with waist circumference ≤ 88 cm. For 5 cm increase in waist circumference at baseline, the risk of CRC increased 8 % in women (HR 1·08, 95 % CI 1·06, 1·10) (Table 2).

### BMI trajectory and risk of colorectal cancer

Table 3 shows the multivariable-adjusted HR of CRC according to different BMI trajectories. Compared with women who kept a relatively low normal BMI throughout adulthood, women who progressed from normal BMI to obesity had a higher risk of CRC (HR 1·29, 95 % CI 1·09, 1·53). Women who progressed from borderline overweight to obesity had a higher risk of CRC (HR 1·37, 95 % CI 1·13, 1·68). Progressing from normal BMI to overweight (HR 1·10, 95 % CI 0·95, 1·27) and kept a relatively high normal BMI (HR 1·14, 95 % CI 0·98, 1·32) were not significantly associated with CRC risk.

**Table 3:** BMI trajectories and the risks of colorectal cancer

Groups	BMI trajectories	Case/population per group	HR	95 % CI*	*P* value
Group 1	Normal to obesity	257/11 789	1·29	1·09, 1·53	< 0·01
Group 2	Normal to overweight	382/19 519	1·10	0·95, 1·27	0·20
Group 3	High normal stable	334/17 660	1·14	0·98, 1·32	0·09
Group 4	Borderline overweight to obesity	152/7241	1·37	1·13, 1·68	< 0·01
Group 5	Low normal stable	386/22 825	reference		

HR, hazard ratio; kg, kilogram; cm, centimetre; NSAID, nonsteroidal anti-inflammatory drugs.

* In Cox proportional hazard regression, adjusted covariates are age, race/ethnicity, education, physical activity, pack-years of smoking, alcohol use, healthy eating index score, family history of CRC, CRC screening, history of colorectal polyp removal, diabetes, prior hormone use and NSAID use.

### Effect modification and sensitivity analyses

For the effect modification analysis, none of the p-values for the interaction of all exposures with physical activity, family history of CRC, education, alcohol consumption, smoking status and hormone therapy were significant (all *P* value > 0·05). Excluding the first 2 years of follow-up, the positive associations among BMI trajectories, BMI at age 50, BMI at baseline, weight change between 18 and 50, waist circumference and the CRC risk are all persistent in multivariable-adjusted model (online Supplementary Table 2 and 3). The positive association between BMI at age 18 and CRC risk was slightly diminished in women with obesity in the multivariable-adjusted model (HR 1·43 95 % CI 0·89, 2·32) compared with women with normal BMI. For considering the possible misclassification of trajectories membership, the association between BMI trajectories and the risk of CRC did not appreciably change after using the posterior probabilities as the exposure (leave the reference group out) (online Supplementary Table 4). Supplementary Table 5 shows that higher waist circumference (> 88 cm) is associated with a 56 % increased risk of colon cancer (HR 1·56, 95 % CI: 1·16, 2·10) compared with those with a smaller waist circumference. Additionally, we found that women who progressed from normal BMI to obesity (HR 1·29, 95 % CI: 1·01, 1·53), as well as those who progressed from borderline overweight to obesity (HR 1·37, 95 % CI: 1·13, 1·68), had a higher risk of colon cancer compared with women with a normal BMI throughout adulthood. However, there was not enough evidence to suggest similar associations for rectal cancer.

## Discussion

In this study, higher BMI at different time points was positively associated with CRC risk among postmenopausal women. Moreover, significant associations between BMI trajectory and CRC risk were observed for women who had a normal weight in early adulthood and progressed to obesity, as well as those remaining overweight /obesity, comparing to women kept a relatively low normal BMI. Excessive weight gain and higher baseline waist circumference were also linked to higher risks of CRC.

Among the prior studies that investigated the association between early adulthood (age ranges: 18–25) BMI and CRC risk in women, five out of seven found non-significant associations^(9–14,23)^. Zhang et al.^(14)^ found a higher risk of CRC in women comparing BMI (at age 18) categories ≥ 27·5 to < 19 kg/m^2^. Han et al^(12)^. found 5 kg/m^2^ increment in BMI (at age 25) was associated with higher risk of CRC in women. The divergent results in previous studies may in part be due to the different study designs, inconsistent anthropometric measures (self-reported or measured) and various adjustments. However, a meta-analysis that pooled individual studies, stratified by sex and identified a positive association between early age (≤ 30 years) BMI and adulthood CRC risk in women^(24)^. Similarly, our study found that the women with obesity at age 18 had a higher risk of CRC, compared with women with normal BMI at age 18. In addition, for 5 kg/m^2^ increase in BMI at age 18, the risk of CRC increased 16 % in women.

Prior studies often employ a single BMI measure, which makes it difficult to disentangle the association of early adulthood obesity from adult obesity due to their strong correlation^(25)^. However, GMM could identify body size patterns through adulthood, and BMI trajectories might help us better understand the cumulative effect of adiposity. We are aware of only one other study that focused on the association between four BMI trajectories (normal BMI, normal BMI to overweight, normal BMI to obese and overweight to obese) and CRC risk combining men and women^(16)^. Zheng et al.^(16)^ found a positive association between the trajectories and CRC risk, observing that individuals who reported being lean in early adulthood and subsequently progressing to obesity in later life had a higher CRC risk, compared with participants who maintained normal BMI. Similar results were also shown in our study which focused on postmenopausal women. In addition, we found that remaining heavy throughout the adult life course also conferred an increased risk of CRC in women.

Whereas BMI depends on both lean and fat mass, weight gain during adulthood is considered as another dynamic metabolic indicator that is more sensitive to an increase in adiposity. Previous sex-stratified studies found non-significant or positive associations between adult weight gain and CRC risk in women^(8–12)^. Besides limited sample sizes, the various definitions of excessive weight gain (range:5·5–24·8 kg) across the studies may explain the inconsistent findings^(8–12,26–28)^. Our study found adult weight gain > 15 kg was associated with an elevated risk of CRC in women. Our results were in line with findings of a recent meta-analysis which found that high body weight gain (midpoint:15·2 kg) increased CRC risk in women^(24)^.

Waist circumference, an indicator for central adiposity, is more consistently associated with CRC risk than other anthropometric measurements. Moreover, studies have reported an independent association between waist circumference and CRC risk after adjustment for BMI^(29,30)^. Unfortunately, we did not have data on long-term waist circumference gain and could not investigate the association between dynamic central adiposity and CRC risk. However, Song et al.^(30)^ examined a 10-year change in waist circumference and its association with CRC risk in the Nurses’ Health Study (NHS) and Health Professionals Follow-up Study (HPFS), finding a positive association in men but not in women.

Although there is convincing evidence linking obesity to colorectal cancer risk, the underlying biological mechanisms have not yet been fully understood. Excess adipose tissue is associated with low-grade inflammation and may produce inflammatory markers including resistin, TNF *α* and IL-6^(31–33)^. Thus, women with overweight or obesity at an early adulthood will be exposed to a longer period of inflammation compared with women with a stable and normal body size^(34)^. In addition, obesity-induced insulin resistance plays a crucial role in the complex metabolic pathways in colorectal carcinogenesis^(35)^. Waist circumference is a sensitive predictor of insulin resistance^(36)^. Moreover, biological evidence shows that waist circumference may be a stronger indicator of insulin resistance than BMI among middle-aged or older adults^(37)^.

Strengths of the current study include the prospective study design, large sample size, sufficient follow-up time, adjudicated cancer cases, a wide range of confounding factors adjustment and the use of multidimensional measures of body size over the adult life course. The current study has several limitations. First, all the pre-baseline weights were self-reported, which is prone to misclassification^(38)^. However, self-reported body size was commonly used and has been validated in previous studies^(39)^. For example, previous study found that the correlations between self-reported and measured weights were as high as 0·97 in the WHI^(39)^. In addition, due to the prospective nature of WHI study, the misclassification is most likely to be nondifferential and lead to an attenuation of the association between body size and CRC. Second, although WHI-OS is a prospective study with a large population and long-term follow-up, we only have limited cases of rectum cancer, especially after stratified by BMI categories. However, colon cancer and rectum cancer differ histologically and physiologically. In our study, colon cancer may drive the association between adult body size and CRC risk, which may not be able to extent to the associations between adult body size and rectum cancer in women. Third, weight loss may happen in the early phase of CRC and is prone to cause possible reverse causality. In our sensitivity analysis, we excluded the first two years of follow-up and found that the positive association between BMI at age 18 and CRC risk was diminished in obesity groups in full model. However, the percentage change of effect size is less than 10 %. Also, the positive association between BMI at age 18 and the CRC risk did not appreciably change for each 5 kg/m^2^ increased in BMI at age 18 in women. In addition, the positive associations among BMI trajectories, BMI at age 50, BMI at baseline, weight change between 18 and 50, waist circumference and the CRC risk are all persistent in multivariable-adjusted model. Fourth, one of the covariates, HEI scores, is computed from FFQ. FFQ is prone to recall bias compared with short-term dietary recording and recall methods. However, one study assessed the validity of WHI FFQ and found that the nutrient estimates obtained from FFQ were similar to those derived from dietary records and recordings^(22)^. Fifth, although GMM is a useful approach to identifying the underlying pattern of body size trajectories, it cannot uncover all the actual developmental patterns. We were not able to differentiate some BMI trajectories, such as weight fluctuation and weight loss. Prior one study did not find a significant association between weight cycling and CRC risk using WHI data^(40)^. Another WHI study found that intentional weight loss, not unintentional weight loss, was associated with lower CRC risk^(41)^.

In conclusion, women who have a normal weight in early adult life and gain substantial weight later, as well as those who are persistently heavy over adulthood demonstrated a higher risk of CRC compared with women kept a normal BMI. Our study highlights that maintaining a healthy body size over the adult life course, especially avoiding early adulthood obesity, might help reduce the risk of developing CRC in women. More research is needed to elucidate how adult body size may influence colon and rectal cancer separately.

## Supplementary material

For supplementary material accompanying this paper visit https://doi.org/10.1017/S1368980023000988. click here to view supplementary material
