# Supplementary material for: Body size over the adult life course and the risk of colorectal cancer among postmenopausal women
Source: Public Health Nutr. 2023 May 18;26(8):1539–48. doi: 10.1017/S1368980023000988 (PMC10410385; doi:10.1017/S1368980023000988)
Supplement: Supplementary file 1 [file S1368980023000988sup001.docx]

Supplemental tables

**Supplemental Table 1. Model comparison indices for different numbers of latent classes**

| **The number of latent classes** | **AIC** | **BIC** | **Entropy** | **Class 1^a^** | **Class 2^a^** | **Class 3^a^** | **Class 4^a^** | **Class 5^a^** |
| --- | --- | --- | --- | --- | --- | --- | --- | --- |
| 2 | 1427651 | 1427902 | 0.690 | 67% | 33% |  |  |  |
| 3 | 1416309 | 1416689 | 0.607 | 16% | 35% | 49% |  |  |
| 4 | 1412204 | 1412487 | 0.554 | 14% | 37% | 31% | 18% |  |
| 5 | 1409486 | 1410127 | 0.537 | 15% | 25% | 22% | 9% | 29% |
| ^a^ The percentage of participants in each class out of the total number of participants  The lower AIC/BIC values indicate better model fit.  Abbreviations: AIC: Akaike Information Criterion; BIC: Bayesian Information Criterion | | | | | | | | |

**Supplemental Table 2: Body size over the adult life course and risk of colorectal cancer excluding 2 years follow-up ^a^**

|  | **Colorectal cancer cases/ No. of participants** | **Age-adjusted**  **HR (95%CI)** | **Multivariable-adjusted ^b^**  **HR (95%CI)** |
| --- | --- | --- | --- |
| **BMI at age 18** |  |  |  |
| Underweight | 200/12,442 | 0.94 (0.81, 1.09) | 0.93 (0.80, 1.09) |
| Normal (reference) | 1,065/60,485 | 1(reference) | 1(reference) |
| Overweight | 65/4,332 | 0.96 (0.75, 1.23) | 0.87 (0.68, 1.12) |
| Obesity | 16/755 | 1.56 (0.97, 2.52) | 1.43 (0.89, 2.32) |
| **Hazard ratio for 5 kg/m^2^ increase** | 1,346/78,014 | **1.16 (1.05, 1.28)** | **1.11 (1.01, 1.23)** |
| **BMI age 35** |  |  |  |
| Underweight | 49/3,623 | 0.79 (0.59, 1.05) | 0.76 (0.57, 1.02) |
| Normal (reference) | 1,114/64,100 | 1(reference) | 1(reference) |
| Overweight | 148/8,326 | 1.17 (0.99, 1.39) | 1.06 (0.89, 1.27) |
| Obesity | 35/1,965 | 1.39 (0.99, 1.93) | 1.21 (0.87, 1.70) |
| **Hazard ratio for 5 kg/m^2^ increase** | 1,346/78,014 | **1.23 (1.13, 1.35)** | **1.17 (1.07, 1.29)** |
| **BMI age 50** |  |  |  |
| Underweight | 27/1,585 | 1.09 (0.74, 1.60) | 1.08 (0.74, 1.59) |
| Normal (reference) | 828/50,974 | 1(reference) | 1(reference) |
| Overweight | 377/18,658 | **1.44 (1.28, 1.63)** | **1.35 (1.19, 1.53)** |
| Obesity | 114/6,797 | **1.48 (1.21, 1.80)** | **1.28 (1.04, 1.57)** |
| **Hazard ratio for 5 kg/m^2^ increase** | 1,346/78,014 | **1.24 (1.16, 1.33)** | **1.17 (1.09, 1.26)** |
| **BMI at baseline** |  |  |  |
| Underweight | 13/941 | 0.97 (0.56, 1.68) | 0.97 (0.56, 1.68) |
| Normal (reference) | 492/31,903 | 1(reference) | 1(reference) |
| Overweight | 471/26,960 | **1.16 (1.02, 1.31)** | 1.08 (0.95, 1.23) |
| Obesity | 370/18,210 | **1.53 (1.34, 1.75)** | **1.33 (1.15, 1.54)** |
| **Hazard ratio for 5 kg/m^2^ increase** | 1,346/78,014 | **1.19 (1.14, 1.25)** | **1.13 (1.07, 1.20)** |
| **Weight change between 18-50^c^** |  |  |  |
| Weight loss <-5kg | 36/2,055 | 1.13 (0.81, 1.58) | 1.10 (0.78, 1.53) |
| Normal (-5kg to 5 kg) (reference) | 464/27,805 | 1(reference) | 1(reference) |
| Weight gain (5kg to 15kg) | 567/32,187 | 1.11 (0.98, 1.26) | 1.07 (0.95, 1.22) |
| Weight gain (>15kg) | 279/15,967 | **1.37 (1.18, 1.59)** | **1.20 (1.04, 1.40)** |
| **Hazard ratio for 5 kg increase** | 1,346/78,014 | **1.08 (1.05, 1.11)** | **1.05 (1.02, 1.09)** |
| **Waist circumference at baseline** |  |  |  |
| Waist circumference ≤88cm (reference) | 812/52,577 | 1(reference) | 1(reference) |
| Waist circumference ＞88cm | 534/25,437 | **1.50 (1.34, 1.67)** | **1.34 (1.19, 1.51)** |
| **Hazard ratio for 5 cm increase** | 1,346/78,014 | **1.10 (1.08, 1.12)** | **1.08 (1.06, 1.11)** |
| ^a^ Cox proportional hazard regression model were used for constructed models. *P* for trend was examined by the continuous of BMI, weight change and waist circumference.  ^b^ In the multivariate-adjusted models, potential confounders include the variables listed in the Covariates section above.  ^c^ Height was further adjusted inweight change model.  Abbreviations: HR: hazard ratio; BMI: body mass index; kg: kilogram; cm; centimetre. | | | |

**Supplemental Table 3. BMI trajectories and the risks of colorectal cancer** **excluding 2 years follow-up**

| **Groups** | **BMI trajectories** | **Case/ population per group** | **HR (95% CI) ^a^** | **P-value** |
| --- | --- | --- | --- | --- |
| Group 1 | Normal to obesity | 227/11,611 | **1.31(1.09, 1.56)** | <0.01 |
| Group 2 | Normal to overweight | 347/19,267 | 1.13(0.97, 1.32) | 0.11 |
| Group 3 | High normal stable | 293/17,439 | 1.13(0.97, 1.33) | 0.12 |
| Group 4 | Borderline overweight to obesity | 134/7,110 | **1.41(1.14, 1.74)** | <0.01 |
| Group 5 | Low normal stable | 345/22,587 | reference |  |
| In Cox proportional hazard regression, adjusted covariates are age, race/ethnicity, education, physical activity, pack-years of smoking, alcohol use, healthy eating index score, family history of CRC, CRC screening, history of colorectal polyp removal, diabetes, prior hormone use, and NSAIDs use.  Abbreviations: HR: hazard ratio; CI: confidence interval; BMI: body mass index. | | | | |

**Supplemental Table 4. BMI trajectories and the risks of colorectal cancer** **using the posterior probabilities as the exposure**

| **Groups** | **BMI trajectories** | **HR (95% CI) ^a^** | **P-value** |
| --- | --- | --- | --- |
| Group 1 | Normal to obesity | **1.42(1.17, 1.81)** | <0.01 |
| Group 2 | Normal to overweight | 1.16(0.90, 1.50) | 0.25 |
| Group 3 | High normal stable | 1.14(0.89, 1.45) | 0.30 |
| Group 4 | Borderline overweight to obesity | **1.61(1.24, 2.09)** | <0.01 |
| Group 5 | Low normal stable | reference |  |
| In Cox proportional hazard regression, adjusted covariates are age, race/ethnicity, education, physical activity, pack-years of smoking, alcohol use, healthy eating index score, family history of CRC, CRC screening, history of colorectal polyp removal, diabetes, prior hormone use, and NSAIDs use.  Abbreviations: HR: hazard ratio; CI: confidence interval; BMI: body mass index. | | | |

**Supplemental Table 5: Waist circumference, BMI trajectories and risk of colon/rectum cancer**

|  | **Cases** | | **Multivariable-adjusted ^a^**  **HR (95%CI)** | |
| --- | --- | --- | --- | --- |
|  | **Colon cancer** | **Rectum cancer** | **Colon cancer** | **Rectum cancer** |
| **Waist circumference at baseline** |  |  |  |  |
| Waist circumference ≤88cm | 768/53,156 | 108/53,156 | 1(Reference) | 1(Reference) |
| Waist circumference ＞88cm | 518/25,878 | 75/25,878 | **1.56 (1.16, 2.10)** | 1.36 (0.98, 1.89) |
| **Hazard ratio for 5 cm increase** |  |  | **1.05 (1.05, 1.07)** | **1.04 (1.01, 1.06)** |
| Group1. Normal to obesity | 217/11,789 | 37/11,789 | **1.29(1.01,1.53)** | 1.32(0.83,2.11) |
| Group2. Normal to overweight | 331/19,519 | 41/19,519 | 1.10(0.95, 1.30) | 0.96(0.63, 1.46) |
| Group3.High normal stable | 288/ 17,660 | 39/17,660 | 1.14(0.98,1.32) | 1.05(0.68,1.60) |
| Group4. Borderline overweight to obesity | 128/7,241 | 16/7,241 | **1.37(1.13,1.68)** | 0.94(0.51,1.76) |
| Group5. Low normal stable | 322/22,825 | 50/22,825 | 1(Reference) |  |
| ^a^ In Cox proportional hazard regression, adjusted covariates are age, race/ethnicity, education, physical activity, pack-years of smoking, alcohol use, healthy eating index score, family history of CRC, CRC screening, history of colorectal polyp removal, diabetes, prior hormone use, and NSAIDs use.  Abbreviations: HR: hazard ratio; CI: confidence interval; BMI: body mass index; kg: kilogram; cm; centimetre. | | | | |

**Supplemental table 6 Variance–covariance matrix for five latent classes**

| **Latent Class 1** | | | |
| --- | --- | --- | --- |
|  | **Intercept** | **Slope of age** | **Slope of age^2^** |
| **Intercept** | 10.048 | -4.543 | 0.561 |
| **Slope of age** |  | 3.490 | -0.435 |
| **Slope of age^2^** |  |  | 0.058 |
| **Latent Class 2** | | | |
|  | **Intercept** | **Slope of age** | **Slope of age^2^** |
| **Intercept** | 12.510 | -4.546 | 0.466 |
| **Slope of age** |  | 2.090 | -0.216 |
| **Slope of age^2^** |  |  | 0.024 |
| **Latent Class 3** | | | |
|  | **Intercept** | **Slope of age** | **Slope of age^2^** |
| **Intercept** | 12.770 | -5.626 | 0.597 |
| **Slope of age** |  | 3.694 | -0.438 |
| **Slope of age^2^** |  |  | 0.060 |
| **Latent Class 4** | | | |
|  | **Intercept** | **Slope of age** | **Slope of age^2^** |
| **Intercept** | 15.411 | -9.512 | 1.298 |
| **Slope of age** |  | 10.197 | -1.541 |
| **Slope of age^2^** |  |  | 0.254 |
| **Latent Class 5** | | | |
|  | **Intercept** | **Slope of age** | **Slope of age^2^** |
| **Intercept** | 3.231 | -0.873 | 0.076 |
| **Slope of age** |  | 0.581 | -0.064 |
| **Slope of age^2^** |  |  | 0.009 |

**Supplemental Table 7. Characteristics of the final class**

| **Groups** | **BMI trajectories** | **Case/ population per group** | **BMI at age 18** | **BMI at age 35** | **BMI at age 50** | **BMI at baseline** |
| --- | --- | --- | --- | --- | --- | --- |
| Group 1 | Normal to obesity | 257/11,789 | 20.35±2.23 | 22.33±2.45 | 26.73±3.69 | 32.56±3.83 |
| Group 2 | Normal to overweight | 382/19,519 | 20.05±1.67 | 21.34±1.63 | 23.54±2.01 | 27.00±2.5 |
| Group 3 | High normal stable | 334/17,660 | 21.55±2.83 | 22.96±2.29 | 24.05±2.78 | 25.57±3.28 |
| Group 4 | Borderline overweight to obesity | 152/7,241 | 24.66±4.01 | 27.83±3.85 | 31.17±4.90 | 34.49±6.53 |
| Group 5 | Low normal stable | 386/22,825 | 19.95±1.45 | 20.62±1.36 | 21.32±1.55 | 22.45±1.94 |
| BMIs are presented by means ± standard deviation  In Cox proportional hazard regression, adjusted covariates are age, race/ethnicity, education, physical activity, pack-years of smoking, alcohol use, healthy eating index score, family history of CRC, CRC screening, history of colorectal polyp removal, diabetes, prior hormone use, and NSAIDs use.  Abbreviations: HR: hazard ratio; CI: confidence interval; BMI: body mass index. | | | | | | |

**GRoLTS checklist**

| GRoLTS checklist item | Yes/No | Additional comments |
| --- | --- | --- |
| 1. Is the metric of time used in the statistical model reported? | Yes | The metric of time is years. |
| 2. Is information presented about the mean and variance of time within a wave? | Yes |  |
| 3a. Is the missing data mechanism reported? | Yes | Mplus assume missing at random (MAR) for variables. |
| 3b. Is a description provided of what variables are related to attrition/missing data? | Yes | Figure 1 |
| 4. Is information about the distribution of the observed variables included? | Yes | Table 1 |
| 5. Is the software mentioned? | Yes | Mplus |
| 6a. Are alternative specifications of within-class heterogeneity considered (e.g., LGCA vs. LGMM) and clearly documented? If not, was sufficient justification provided as to eliminate certain specifications from consideration? | Yes | LGCA assumes no variance in the growth parameters within each group, which may not be realistic. However, 5 group BMI trajectory by using LGCA is present in Supplemental Figure1 |
| 6b. Are alternative specifications of the between-class differences in variance–covariance matrix structure considered and clearly documented? If not, was sufficient justification provided as to eliminate certain specifications from consideration? | Yes | Supplemental table 6 |
| 7. Are alternative shape/functional forms of the trajectories described? | Yes | Both linear and quadratic trajectories were considered |
| 8. If covariates have been used, can analyses still be replicated? | NA | The data is not publically available. |
| 9. Is information reported about the number of random start values and final iterations included? | Yes | There are of 1000 initial random start values and maximum iterations is 500. |
| 10. Are the model comparison (and selection) tools described from a statistical perspective? | Yes | Supplemental table 1 |
| 11. Are the total number of fitted models reported, including a one-class solution? | Yes | Supplemental table 1 |
| 12. Are the number of cases per class reported for each model (absolute sample size, or proportion)? | Yes | Supplemental table 1 |
| 13. If classification of cases in a trajectory is the goal, is entropy reported? | Yes | Supplemental table 1 |
| 14a. Is a plot included with the estimated mean trajectories of the final solution? | Yes | Figure 2 |
| 14b. Are plots included with the estimated mean trajectories for each model? | Yes | We present the final 5 BMI groups (Figure 2), as well as the alternative 4 BMI trajectory in Supplemental Figure 2 |
| 14c. Is a plot included of the combination of estimated means of the final model and the observed individual trajectories split out for each latent class? | Yes | It is infeasible to plot all observed individual trajectories split out for each latent class for the given sample size. However, we randomly selected 100 participants and plot their observed individual trajectories in Supplemental Figure3 |
| 15. Are characteristics of the final class solution numerically described (i.e., means, SD/SE, n, CI, etc.)? | Yes | Supplemental table 7 |
| 16. Are the syntax files available (either in the appendix, supplementary materials, or from the authors)? | Yes | Syntax are available upon request from authors. |

**Figures**

**Supplementary Figure 1. Five groups body mass index trajectories using latent class growth analysis**

**Supplementary Figure 2. Four groups body mass index trajectories using growth mixture model**


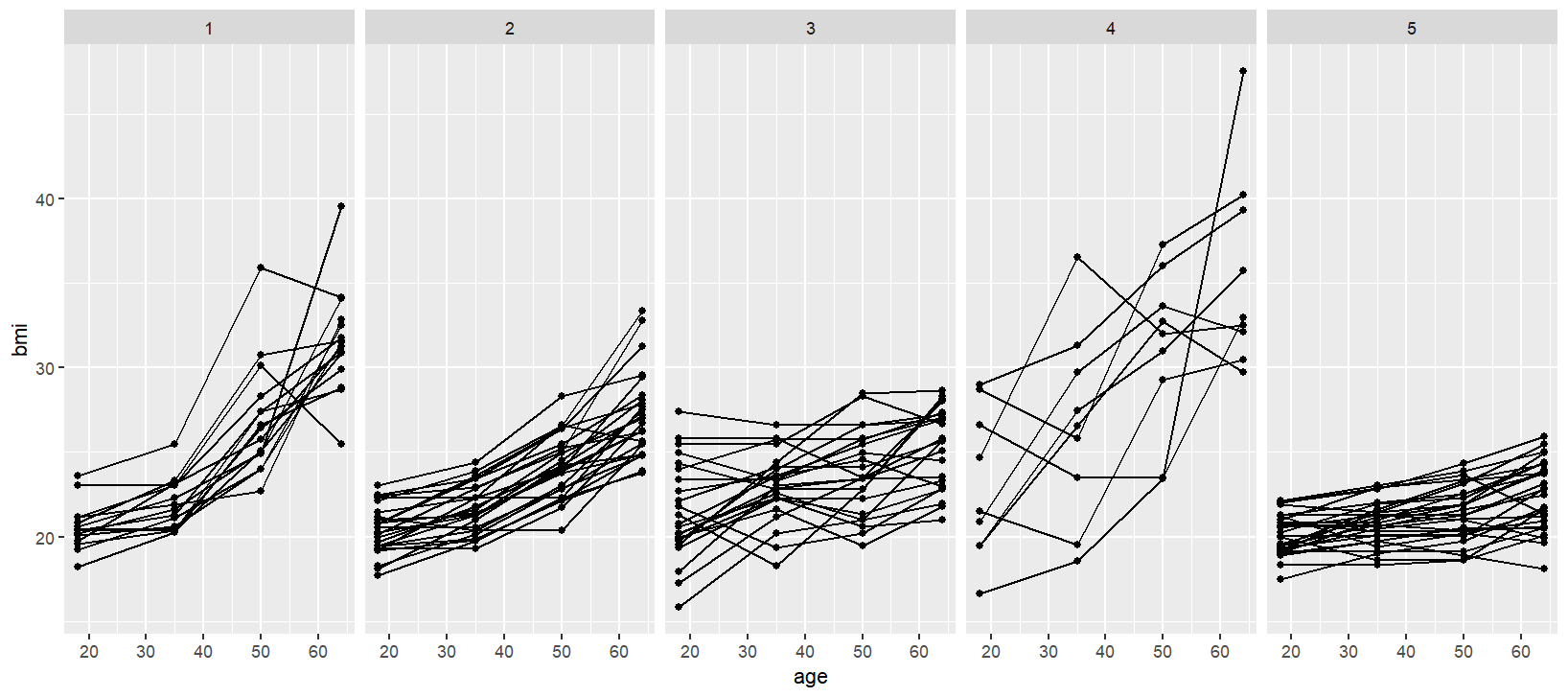


**Supplementary Figure 3 observed individual trajectories split out for each latent class**

**SHORT LIST OF WHI INVESTIGATORS**

**Program Office:** (National Heart, Lung, and Blood Institute, Bethesda, Maryland) Jacques Rossouw, Shari Ludlam, Joan McGowan, Leslie Ford, and Nancy Geller

**Clinical Coordinating Center:** (Fred Hutchinson Cancer Research Center, Seattle, WA) Garnet Anderson, Ross Prentice, Andrea LaCroix, and Charles Kooperberg

**Investigators and Academic Centers:** (Brigham and Women's Hospital, Harvard Medical School, Boston, MA) JoAnn E. Manson; (MedStar Health Research Institute/Howard University, Washington, DC) Barbara V. Howard; (Stanford Prevention Research Center, Stanford, CA) Marcia L. Stefanick; (The Ohio State University, Columbus, OH) Rebecca Jackson; (University of Arizona, Tucson/Phoenix, AZ) Cynthia A. Thomson; (University at Buffalo, Buffalo, NY) Jean Wactawski-Wende; (University of Florida, Gainesville/Jacksonville, FL) Marian Limacher; (University of Iowa, Iowa City/Davenport, IA) Jennifer Robinson; (University of Pittsburgh, Pittsburgh, PA) Lewis Kuller; (Wake Forest University School of Medicine, Winston-Salem, NC) Sally Shumaker; (University of Nevada, Reno, NV) Robert Brunner

**Women’s Health Initiative Memory Study:** (Wake Forest University School of Medicine, Winston-Salem, NC) Mark Espeland

For a list of all the investigators who have contributed to WHI science, please visit: https://s3- us-west-2.amazonaws.com/www-whi-org/wp-content/uploads/WHI-Investigator-Long-List.pdf
